# Wild Fauna in Oman: Foot-and-Mouth Disease Outbreak in Arabyan Oryx (*Oryx leucorix*)

**DOI:** 10.3390/ani15162389

**Published:** 2025-08-14

**Authors:** Massimo Giangaspero, Salah Al Mahdhouri, Sultan Al Bulushi, Metaab K. Al-Ghafri

**Affiliations:** 1 Faculty of Veterinary Medicine, University of Teramo, 64100 Teramo, Italy; 2 Parliamentary Assembly of the Mediterranean, Center for Global Studies, 47890 City of San Marino, San Marino; 3 Environment Authority, Muscat 100, Oman

**Keywords:** Arabian Oryx, biosecurity, foot-and-mouth disease, reintroduction, vaccination

## Abstract

The Sultanate of Oman boasts remarkable biodiversity, exemplified by such species as the Arabian leopard (*Panthera pardus nimr*) and the Arabian oryx (*Oryx leucoryx*), national symbols that highlight the extensive conservation efforts required to protect the country’s natural heritage. During decades, Omani authorities have taken significant measures to safeguard wildlife and preserve the natural environment. A sanctuary dedicated to the reintroduction of the Arabian Oryx, after extinction in nature in 1972, was established in 1980 in the Al Wusta Governorate under the patronage of the Royal Diwan and currently administrated by the recently established Environment Authority. During the almost 40 years since the reintroduction and the creation of the sanctuary, the oryx population has grown slowly but constantly. In 2021, the sanctuary hosted 738 oryx, allowing the start of the reintroduction of the species into the natural environment. Small groups of animals were released into the wild in selected areas. No animal health adverse events were recorded, and mortality was generally due to injuries received as a consequence of fighting, in particular during mating season. Standard veterinary care, including control of internal and external parasites, was regularly provided. In some occasions, immunization against certain diseases, such as clostridial infections, pasteurellosis, or mycoplasmosis, was also applied. In 2023, an FMD outbreak in cattle reported in Dhofar, about 500 km from the Al Wusta sanctuary, motivated specific prophylactic actions to prevent the risk of diffusion to oryx. From December 2023 to January 2024, an immunization program was undertaken using an FMD vaccine against serotypes A, O, and SAT 1, mostly in male oryx, while pregnant oryx were avoided for abortion risk due to handling. The following year, in January 2025, a severe outbreak occurred in oryx herds held in the sanctuary. The rapid onset and the spread of clinical symptoms among animals (100% morbidity in the second day after the first appearance of signs in some individuals) were suggestive of a highly contagious disease. The animals suffered from severe depression and inappetence, rapidly followed by abundant salivation, erosions of the oral mucosa and tongue, and diarrhea, with a short course characterized by prostration and death of the animal in the most severe cases. Therapeutical attempts (administration of antibiotics and rehydration) were mostly ineffective. Laboratory investigations (ELISA and PCR) ruled out contagious bovine pleuropneumonia (CBPP), Johne’s disease and Peste des petits ruminants (PPR). Both serology and antigen detection showed positiveness to foot-and-mouth disease (FMD). Out of a total population of 669 present in the sanctuary at the beginning of the outbreak, 226 (33.78%) oryx died. Despite the vaccinal status, the 38.49% of dead animals resulted being vaccinated against FMD. Taking into account the incalculable value of the species, the outbreak represented a very dangerous event that risked wiping out the decades of conservation efforts. Therefore, all the available means, such as accrued biosecurity and adequate prophylaxis, should be implemented to prevent the recurrence of such health risks. The delicate equilibrium of wild fauna in Oman requires study and support for an effective protection, in line with the national plan “Vision 2040”, targeting the inclusion of the Sultanate within the 20 best virtuous countries for wildlife protection.

## Simple Summary

The Arabian oryx (*Oryx leucoryx*), a national symbol of Oman, was reintroduced in 1980 through a sanctuary in Al Wusta Governorate after its extinction in the wild in 1972. Managed by the Environment Authority, the population grew steadily, reaching 738 individuals by 2021, with no major health issues reported, and enabling limited rewilding efforts. Following a foot-and-mouth disease (FMD) outbreak in cattle, a vaccination program targeting serotypes A, O, and SAT 1 was applied, mainly to male oryx, from December 2023 to January 2024. In January 2025, a severe FMD outbreak struck the sanctuary. The disease spread rapidly (100% morbidity within two days), causing depression, inappetence, oral lesions, diarrhea, and high fatality rates. Treatment attempts were largely ineffective. Laboratory tests confirmed FMD, excluding other diseases. Of 669 oryx, 226 (33.8%) died, including 38.5% that had been vaccinated. This outbreak posed a major threat to decades of conservation progress. Enhanced biosecurity, vaccination strategies, and ongoing ecological research are essential to safeguard Oman’s unique biodiversity.

## 1. Introduction

The Sultanate of Oman, with its vast arid deserts and rugged mountain ranges, is home to an unexpectedly rich variety of remarkable wildlife. Among its most striking and iconic species is the majestic Arabian oryx or white oryx (*Oryx leucoryx*, Pallas, 1777). Four other species of oryx exist: the scimitar-horned oryx (*Oryx dammah*, Cretzschmar, 1827), the East African oryx (*Oryx beisa*, Rüppell, 1835), the fringed-eared oryx (*Oryx beisa callotis*, Thomas, 1892), and the Gemsbok (*Oryx gazella*, Linnaeus, 1758). The Arabian species is the smallest species of oryx (size 1.53–2.35 mt; weight 65–70 kg), able to live in most hostile conditions, perfectly adapted to a dry and arid environment, with white hair, very low perspiration and moisture loss, and a peculiar physiology allowing partial cooling of the brain and rise of body temperature up to 45 degrees without suffering [1].

The Arabian oryx (*Oryx leucoryx*) was extinct in the wild 50 years ago, mainly due to uncontrolled poaching [2]. Fortunately, the reintroduction was possible, at least in captivity, in some Arabian countries. Currently, only a few individuals are in the wild, and about 6,000 live in sanctuaries [3]. In Oman, for the purpose of reintroducing the Arabian oryx (*Oryx leucoryx*), in 1980, the Oryx Sanctuary was created, the first wildlife reserve and still the most relevant protected area in the country [2]. The sanctuary is located within the central desert and coastal hills in the Al Wusta Governorate, an area characterized by seasonal atmospheric conditions that support a unique desert ecosystem [4]. Since the global extinction in 1972, ten years after the death of the last oryx in Oman, the first animals born in American zoos could be reintroduced here in 1982 [2]. In 1994, the sanctuary was inscribed in the list of the United Nations Educational Scientific and Cultural Organization (UNESCO) World Heritage Centers (Dossier number 654), based on the criterium of a property of 2,750,000 ha, containing the most important and significant natural habitats for in situ conservation of biological diversity, including those containing threatened species of outstanding universal value from the point of view of science or conservation [5]. However, due to hydrocarbon exploitation, the protected area was drastically reduced by 90%, inducing UNESCO to delist the sanctuary in 2007 [6].

Since the first reintroduction of the animals, in a few decades, their number increased at the Al Wusta Sanctuary, reaching even good populations with over 900 individuals. This increase and the apparent absence of health problems allowed the start of the reintroduction of the species in the natural environment and small groups of animals have been released into the wild in selected areas. In addition, other species were introduced in the reserve, such as the Arabian sand gazelle (*Gazella marica*, Thomas, 1897), also known as reem gazelle. The sand gazelle was particularly prolific, and in 2021, the sanctuary hosted over 1150 Arabian sand gazelles, along with 738 Arabian oryx.

During the almost forty years of activity, no animal health adverse events were recorded, and mortality was generally due to injuries suffered as a consequence of fighting, particularly during mating season [2,7]. Standard veterinary care, including control of internal and external parasites, was regularly provided. On some occasions, immunization against certain diseases, such as clostridial infections, pasteurellosis, or mycoplasmosis, was also applied. Animals were fed with 55–70 kg of hay, 10 kg of lucerne, and 50 kg of concentrate daily. Fresh drinking water and salt blocks were available for the animals.

### 1.1. FMD in Oman

The infection was reported as present in the domestic animals in the country almost continuously. In the World Organization for Animal Health (WOAH) databases, the disease was declared to occur before 2005 and in the WHAIS since 2005, with two exceptions: absent during the second semester 2019 and the first semester 2024 and again present in the second semester 2024. However, concerning wild animals, FMD was reported absent until 2008, and later until 2024, no information was provided to WOAH [8].

### 1.2. The FMD Outbreak in Cattle in Dhofar

Infection with foot-and-mouth disease virus was formally reported to the World Organisation for Animal Health on the 1st of January 2023, under event ID number 5178. By immediate notification, as FMD is a listed disease, the Ministry of Agriculture of the Sultanate of Oman declared an official occurrence of FMD among domestic cattle from herds located near Salalah, in the southwestern administrative region of Dhofar. The reported cases were 5,100 and the deaths accounted for 23 bovines. By the vaccination of 5,100 heads, the outbreak was resolved and closed on the 28th of January 2023. The reason for notification was indicated as a new strain in the country and the virus was characterized as subtype SAT 2 by the analysis performed at the reference laboratory of Pirbright, UK, confirming the FMD event on the 28th of March 2023. This was the last notification from Oman to the WOAH (OIE) recorded in the WHAIS.

Currently, a prophylaxis program against FMD is undertaken at the country level for domestic animals, covering the 60% of cattle and sheep and goat populations, excluding dromedary camels. A vaccine against FMDV serotypes O, A, SAT 2, and Asia 1 was selected for the immunization of animals. Not all serotypes were considered for economic reasons, and serotype C is very rare and never reported in the country. FMDV serotype C has not been isolated by the network of WOAH/FAO reference laboratories for FMD since 2004, and no serotype C events have been reported to WOAH since then [8].

### 1.3. Extended Vaccination in Wildlife (Before Outbreak)

Considering that the FMD outbreak occurred in cattle from Dhofar, about 500 km from the Al Wusta sanctuary, to prevent the risk of virus diffusion, specific prophylactic actions were extended to the Arabian oryx. The FMD vaccination plan in Arabian oryx in Al Wusta was similar to the national program, but restricted to serotypes A, O, and SAT 1. From December 2023 to January 2024, mainly male oryx were immunized, while, being in the middle of reproduction season, most of the females were excluded due to advanced pregnancy and effective risk of abortion if handled. However, the number of animals was consistent and in total, 465 oryx were vaccinated, including 102 females.

### 1.4. The FMD Outbreak in Oryx Population in Al Wusta

During about four weeks, from the 8 January to the 4 February 2025, a severe outbreak occurred in the Arabian oryx herds held in the sanctuary of Al Wusta. The rapid onset and the spread of clinical symptoms among animals (100% morbidity in the second day after the first appearance of signs in some individuals) were suggestive of a highly contagious disease. The animals suffered from severe depression and inappetence, rapidly followed by abundant salivation, erosions of the oral mucosa and tongue, and diarrhea, with a short course characterized by prostration and death of the animal in the most severe cases.

First, therapeutic attempts were applied. Treatments consisted of the administration of water soluble powder of oxytetracycline (Spectra cycline, Al Mutawwaj Veterinary Pharmaceutical Industries, Amman, Jordan) 1 g per 10 kg body weight (bw) for 3–6 days; oral administration of florfenicol (Introflor-100 oral, Holland, Viimsi, Estonia) 10 mg/kg bw in drinking water for 5 days; oral administration of gentamycin and tylosin association (Genta-Tylan, Travetco, Can Tho, Vietnam) 1 g/5 kg bw, continuous use for 3–5 days; injectable solutions of tylosine (Hangen-Tylo, Hanvet, Hanoi, Vietnam) via intra muscular (IM) injection 10 mL/50 kg bw, twice a day; selenium and vitamin E (Selephos, Topkim, Istambul, Turkey) 4 ml IM, 3–4 injections within 1 week; and non-steroidal anti-inflammatory drug containing ketoprofen, (Ketovet, Mobedco-Vet, Amman, Jordan) 3 mg/kg IM bw, once daily for up to 3–5 days and intravenous rehydration with saline solutions. In oryx, therapies did not provide evident beneficial effects, and in certain cases, it was not possible to administrate any medicinal product since the clinical course was too rapid and with fatal outcome.

Laboratory investigations have been conducted to ascertain the cause of the disease affecting the entire oryx population. Among different considered pathogens, laboratory tests confirmed only the presence of foot-and-mouth disease (FMD).

The other wild animal species present in the sanctuary were almost not affected by the ongoing outbreak in oryx. Out of the over 1150 sand gazelles (*Gazella marica*, Thomas, 1897) held in the Al Wusta natural reserve, only 20 cases with 4 deaths were reported. In this species, clinical cases were treated with doxycycline and tylosine administered per os by drinking water for 3 days, resulting in recovery. However, among female reems at full-term pregnancy, an increased fetal mortality was observed, with subsequent difficult delivery and mother death. Among Arabian gazelles (*Gazella arabica*, Lichtenstein, 1827) and Arabian mountain gazelles (*Gazella gazella cora*), only 1 fatal case was recorded in each species. Mortality was also reported in one blackbuck (*Antilope cervicapra*), also known as Indian antelope. In the ten llama (*Lama glama*, Linnaeus, 1758), living with the oryx, no clinical sings of disease were observed, despite new world camelids having been shown to be receptive to FMD infection [9].

The present study aimed to provide details of the FMD outbreak that occurred in Arabian oryx, the most deadly event never reported before in the species since its reintroduction from extinction in the wild. Taking into account the value of the wild fauna in the Sultanate, the overview of management difficulties related to breeding captive wild species is indicative of necessary conservation efforts and future prospects to address threats and challenges and protect animal health from epidemiological risks, with particular attention to the Arabian oryx and other vulnerable and endangered species.

## 2. Material and Methods

### 2.1. Animals

The Arabian oryx population was distributed in four different herds at the Al Wusta Sanctuary, Sultanate of Oman, accounting for a total of 669 individuals, 657 adults (312 males and 345 females), and 12 calves, recorded present on the 1 January 2025 (Table 1). As recorded in the animal register of the sanctuary and based on the monthly census performed by the staff, the animals were subdivided in 4 herds, held in four different enclosures, indicated as maha 1, 2, and 3 (Arabic female given name meaning half moon or beautiful eyes), and the fourth as HP. The herd in maha 1 was constituted only by adult males (n 163), while the other herds were mixed with both adult males and females (maha 2: M 53, F 171, and 8 calves; maha 3: M 46, F 144, and 4 calves; and HP: M 50 F 30).

Vaccinal campaign against FMD applied on Arabian oryx population, from the 26 December 2023 to the 22 January 2024, following the reported outbreak in cattle that occurred in Dhofar in January 2023 (Table 2). Each animal, a total of 465 individuals (M 363, F 102), was immunized with 2 mL subcutaneously (SC) with a polyvalent inactivated vaccine against FMD serotypes O, A, and SAT 2, commercialized for cattle, sheep, and goats (Vet. Ser. and Vacc. Res. Inst., Cairo, Egypt). One hundred vaccinated males were released into the wild from January 2024 to January 2025. The FMD vaccine was systematically administered simultaneously with a vaccine against enterotoxemia or hemorrhagic septicemia (Multiclostri 12P, Vac Sules, Canelones, Uruguay). Other animals were not protected for FMD, or immunized against other diseases (n 52) (clostridial infections, pasteurellosis, or mycoplasmosis), nor not immunized, especially pregnant females, to avoid risk of abortion due to handling.

### 2.2. Monitoring of the Animals

Clinical examinations of animals showing abnormal behavior or apparent signs of illness were constantly performed by the staff to describe symptoms and eventual lesions. Morbidity and mortality rates were recorded daily. Some dead animals were necropsied to report gross lesions affecting internal organs. Due to the amplitude of the outbreak, implying relatively high numbers of deaths, and the lack of facilities available for use as a necropsy room and refrigerated chambers for carcasses, autopsies were conducted directly on the field and limited to approximative observations.

### 2.3. Samples

Sixteen samples were collected from clinically affected animals, 15 Arabian oryx and 1 sand gazelle (reem). Arabian oryx: twelve females, ear tag numbers 0094, 0098, 0447, 0464, 0500, 0994, 3, 245, 400, 405, 413, and 472; three males, ear tag numbers 83, 140, and one untagged; and sand gazelle: one untagged male.

Blood samples, collected from jugular vein in EDTA or silicone 10 mL tubes, and fresh feces, directly collected from the anus, were placed in an ice box and transported to the Central Laboratory for Animal Health, Directorate General of Animal Wealth, Ministry of Agricultural Wealth, Fisheries and Water Resources, Muscat, Sultanate of Oman. The tests were performed at the Biochemistry, Immunology, and Biotechnology sections of the Central Laboratory for Animal Health.

The first samples were collected on the 13th of January 2025, for complete blood count (CBC), kidney function test (KFT), liver function test (LFT), minerals, and parasitological screening. On the 15th of January, additional samples were collected for serological and antigen investigations.

### 2.4. Laboratory Tests

Hematology and hematochemistry

Complete blood count (CBC) analysis of blood samples from four oryx females (ear tags 0994, 400, 405, and 472) and one male (untagged) was achieved using an automated hematology analyzer (Sysmex XR-1000, Sysmex Corporation, Kobe, Japan). Red blood cells (RBC), white blood cells (WBC), and platelets (PLT) were counted with related parameters: mean corpuscular volume (MCV), nucleated RBC (NRBC), red cell distribution width standard deviation (RDW-SD), red cell distribution width coefficient of variations (RDW-CV), mean platelet volume (MPV), and platelet large cell ratio (P-LCR). Hemoglobin (Hb), mean corpuscular hemoglobin (MCH), corpuscular hemoglobin concentration (MCHC), as well as hematocrit (HCT) and procalcitonin (PCT) values were also obtained.

Blood samples for the analysis of biochemical parameters for a kidney function test (KFT), liver function test (LFT), and minerals were centrifuged at 2500× *g* for 15 min. Serum and plasma were transferred into polypropylene micro-centrifuge tubes, which were stored at −80 °C until analysis. Serum ions, calcium, phosphorus, magnesium, copper, and iron, and a range of biochemical parameters, including serum albumin, alkaline phosphatase, bilirubin, creatinine, creatine kinase with N-acetyl cysteine (CK NAK), glucose, and urea, were analyzed with an automatic biochemistry analyzer (Chem 200, Gesan Production, Campobello di Mazara, Trapani, Italy) using absorbance photometry (enzymes and substrates). Values and means for various hematological and serum biochemical parameters were compared to values reported from apparently healthy animals referred to as normal reference limits [10,11,12].

Parasitology

Fecal samples were freshly collected directly from the rectal ampulla of five individual oryx (four females identified with ear tags 0094, 400, 405 and 472 and one untagged male), to avoid contamination by eggs from the environment, using a plastic device for lambs. Three grams of feces from each animal were dissolved in water-saturated NaCl solution at room temperature (20 °C) to recover the eggs of gastrointestinal helminth parasites. Eggs were identified under light microscope.

Serology and Antigenic detection

Serum samples collected from 12 Arabian oryx (7 females, ear tags 0098, 0447, 0500, 0994, 3, 400, 405, 413, and 472, and 3 males, 2 identified with ear tags 83 and 140 and 1 untagged) were tested using commercial kits for antibodies against contagious bovine pleuropneumonia (CBPP) using the latex agglutination test (LAT), and for antibodies against FMDV non-structural proteins (NSP), Johne’s disease (para-tuberculosis) and Peste des petits ruminants virus (PPRV), using enzyme linked immunosorbent assay (ELISA). Taking into account the applied vaccination against FMD in the oryx herd, discriminate infected from vaccinated animals (DIVA) diagnostic testing was used to detect the FMD infection/virus circulation in the vaccinated population. To assess exposure to FMDV during the outbreak in the captive Arabian oryx (*Oryx leucoryx*) population, serum samples were analyzed using the ID Screen^®^ FMD NSP Competition ELISA kit (Innovative Diagnostics, Grabels, France), which is able to detect non capsid protein (3ABC NSP) antibodies in serum and plasma from all FMD-susceptible species. The ELISA was executed with coated 96-well flat-bottom microplates. Optical density (OD) reading was performed using a spectrophotometer at 450 nm (Microplate Reader 680, Bio-Rad, Hercules, CA, USA).

Real-time reverse transcription polymerase chain reaction (RT-qPCR) assay for FMDV and PPRV analysis was conducted on oral swabs collected from 9 Arabian oryx (7 females, ear tags 0098, 0447, 0464, 0500, 3, 245, and 413 and 2 males ear tags 83 and 140) and 1 sand gazelle (untagged male), using commercial test kits (FMDV dtec-RT-qPCR and PPRV dtec-RT-qPCR, Genetic analysis strategies SL, Orihuela, Spain). FMDV-specific RNA was detected targeting the conserved 3D RNA-dependent RNA polymerase gene of the virus. The tests were conducted following manufacturer instructions using a PCR thermal cycler (Mastercycler nexus X2, Eppendorf, Enfield, CT, USA).

## 3. Results

**Figure 1:**
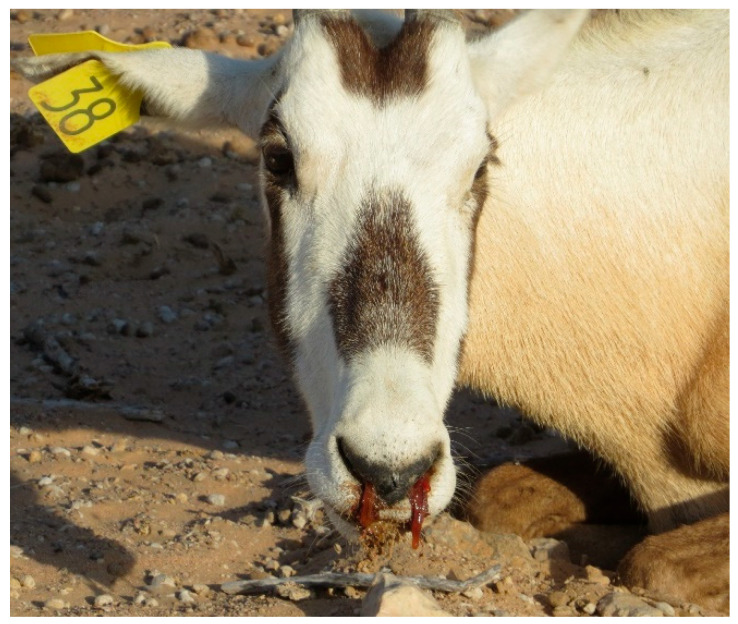
Arabian oryx (*Oryx leucoryx*) at Al Wusta natural reserve showing epistaxis.

**Figure 2:**
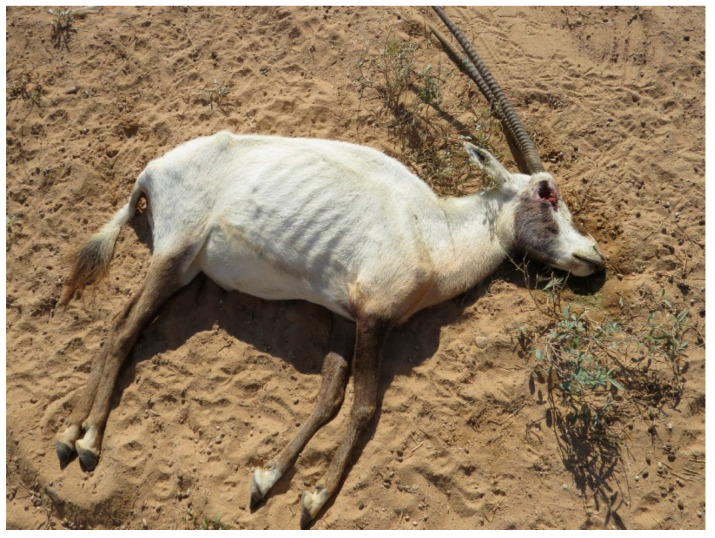
Dead Arabian oryx in poor body conditions. Ocular lesions are due to scavenger birds.

**Figure 3:**
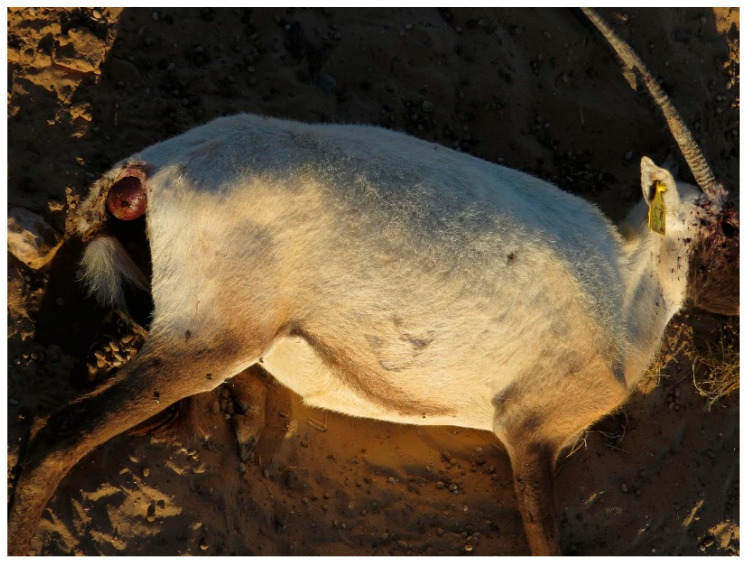
Arabian oryx female dead during delivery.

**Figure 4:**
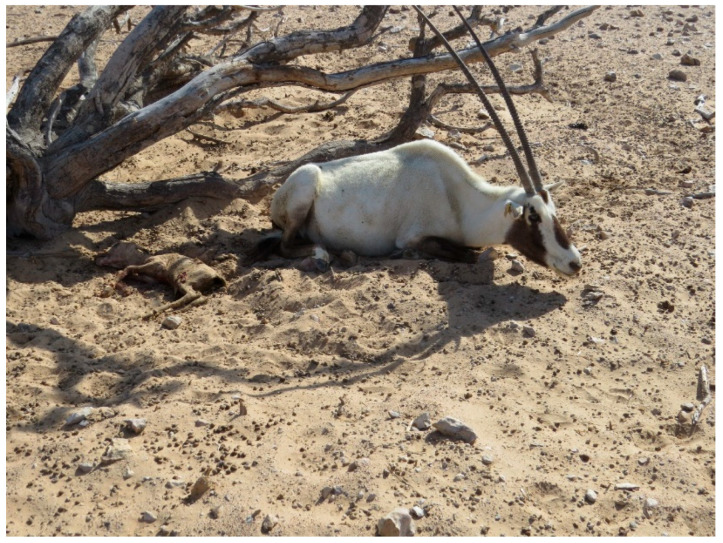
Stillbirth in Arabian oryx.

The monitoring of the Arabian oryx herds showed that clinical symptoms appeared first among the animals present in the maha 1 enclosure out of four, then rapidly diffused in the other three herds located in the other enclosures. The first sign of illness was inappetence. On the first day of the outbreak (the 8th of January 2025), the 25% of oryx showed inappetence. Then, on the third day, all other animals showed the same behavior, indicating a morbidity rate of 100%. Rapidly, other symptoms could be observed. Animals showed asthenia, reluctancy to move, preferring lying on the ground, and often acquiring abnormal postures, with head and neck turned backwards along the flank or extended limbs. Clinical symptoms were characterized by drooling (abundant salivation), limping (abnormal asymmetric gate), watery diarrhea (in some cases with mucus or blood-tinged), tympanism (inducing animals to kick their flanks), vomiting, in some cases bleeding from nostrils (Figure 1), and prostration and death in more severe cases (Figure 2). The severity of the clinical course was high among pregnant and calves, causing mortality among adults, especially at term of pregnancy (Figure 3), stillbirth (Figure 4), or early death of newborns, which was relatively high in other adult females and less severe in adult males.

**Figure 5:**
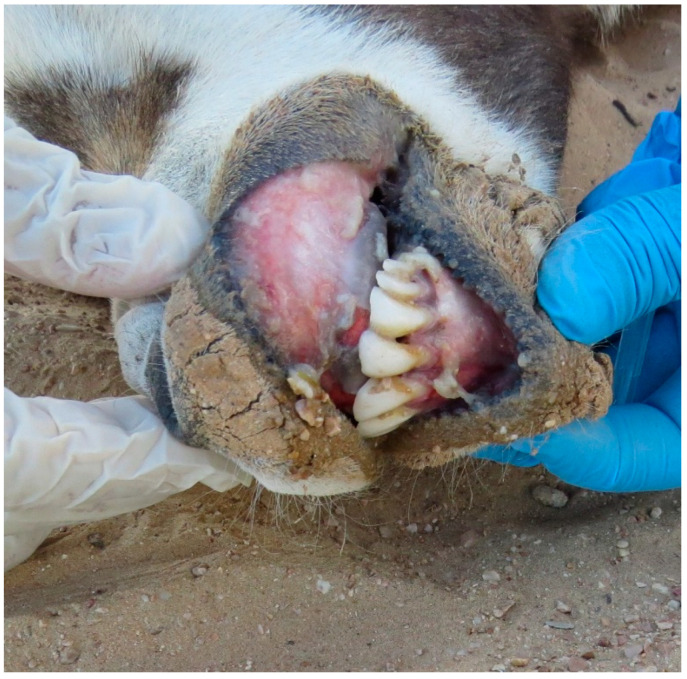
Erosions of gingiva with deposits of necrotic caseous material in Arabian oryx.

**Figure 6:**
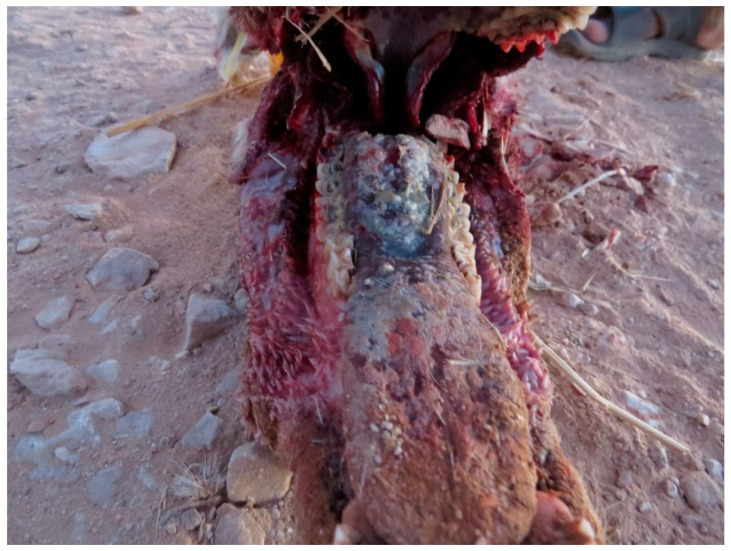
Severe erosions of the tongue and posterior necrotic caseous deposits in Arabian oryx.

**Figure 7:**
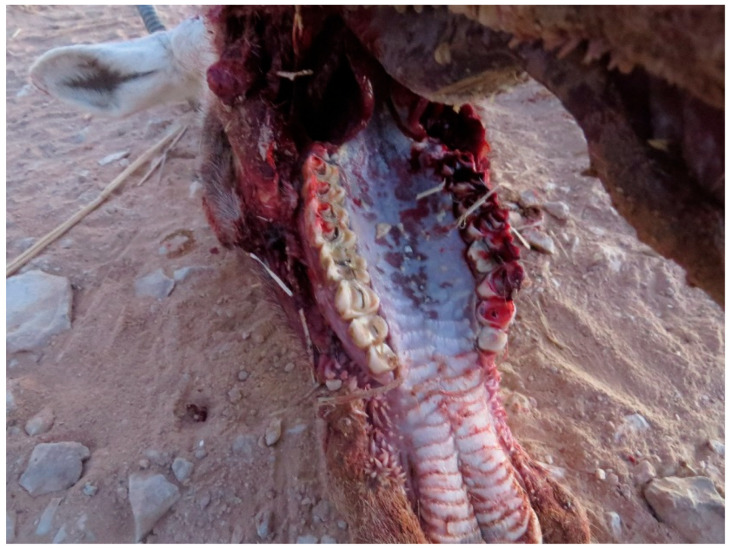
Erosions of the palate in Arabian oryx.

**Figure 8:**
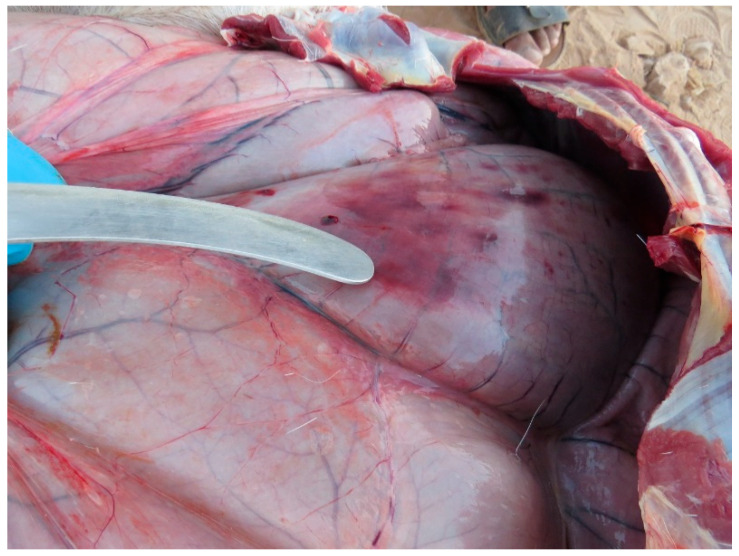
Gastrointestinal tract of Arabian oryx, distended by accumulation of gas and presenting hemorrhages on the serosa.

Lesions consisted in aphthae appearing in the oral cavity and evolving in erosive lesions. The erosions were observed on the gum of the upper lip, gingival mucosa (Figure 5), including peri dental gingiva, tongue (upper epithelium and lower mucosa) (Figure 6), and palate being eventually covered by necrotic caseous material (Figure 7). No animals showed lesions at the level of coronary band. The gastrointestinal tract was distended by accumulation of gas and presented serosal hemorrhages (Figure 8) and in some cases deposits of fibrinous exudate.

The mortality rate observed among animals held at the Al Wusta sanctuary, during the outbreak from the 8 January to the 4 February 2025, was high, particularly in the Arabian oryx population. The total death toll among Arabian oryx accounted for 226 animals, 82 males and 118 females, and another 26 animals for which gender was not recorded. This was equivalent to 33.78% of the entire population out of 669 animals present in the sanctuary at the beginning of the outbreak (as recorded on the 1 January 2025). With exception made for 24 untagged animals (6 males, 17 females, and 1 calf sex not reported), the majority of the dead oryx were identified by ear tag numbers, and most were documented individually by photos. Pregnancy and poor body conditions (malnutrition) appeared to be risk factors. Mortality was recorded also among vaccinated animals against FMD (n 87, 65 males, and 22 females, 38.49% of dead animals). Details on the vaccinal status of dead animals are given in Table 3. Observations in other species present in the sanctuary (reems, other gazelle species, blackbuck, and llamas) recorded fatal cases in four reems and one blackbuck. Details on mortality in animals are available in Supplementary Material Table S1.

The hematological profile of the animals, evaluated by CBC, KFT, and LFT, at the beginning of the outbreak, showed altered values, on the whole characteristic of a severe disease occurring. All considered, animals suffered from marked leucopenia (white blood cell count mean 6.13) with very low values, even lower than 4 × 10^3^/µL, compared to the normal range from 11 to 14. Neutrophils were severely depleted, with an observed mean of 5.36%, instead of a normal range of 56.5–75.2%, while lymphocytes were augmented, showing a mean of 88.06% (normal range 20.5–28.4%), characteristic of an inflammatory process. Low values of red blood cells, hemoglobin, and platelets were also observed.

High values of bilirubin (mean 0.94 mg/dL, normal range 0.008–0.01), transaminases (GOT mean 118.58 U/L, GPT mean 59.68 U/L, compared to normal ranges of 47–63 and 35–45, respectively), and urea (mean 71.22 mg/dL, normal range 20–25) were consistent with acute hepato-renal sufferance. Additionally, elevated creatine kinase levels (mean 791.48, normal range 303–367) were indicative of kidney failure, in addition to underlying possible muscle damage or hearth issues, as well as the observed hypoalbuminemia (mean 3.24 g/dL, normal range 4.5–5.2) that could be related to nephrotic syndrome or extensive hepatic damage, while hypoglycemia (mean 83.23 mg/dL, normal range 119–150) was probably an expression of the starvation of the animals. The detailed results of the hematological and serum biochemical analyses are reported in Table 4.

The parasitological investigation was performed on fecal samples from Arabian oryx females identified by ear tags 0094, 400, and 405, and one untagged male, showing positiveness for *Nematodirus* spp. eggs (samples 0094 and 400 were highly positive). Females 0094 and 400 were also positive for *Eimeria* spp. oocysts. The sample from female 472 was negative for internal parasites.

The results of the serological and antigenic analyses are reported in Table 5. All serum samples collected from Arabian oryx (females 0994, 400, 405, and 472 and untagged male) were negative for antibodies against *Mycoplasma mycoides* subsp. *mycoides* small colonies, the causative agent of CBPP. Samples from Arabian oryx females (0098 and 413) and males (83 and 140) were negative for antibodies against *Mycobacterium avium paratuberculosis* (Johne’s disease, para-tuberculosis), PPRV, and FMDV-NSP antibodies. The sample collected from Arabian oryx female, identified with ear tag number 3, was the only positive for anti-FMDV-NSP antibodies. The same sample showed a doubtful seroconversion against PPRV; thus, a request for a new sample after 2 weeks was formulated by the laboratory investigators, but the animal died before. Samples collected from two females (ear tags 0447 and 0500) were invalid for serological testing due to hemolysis. Blood samples obtained from six Arabian oryx females (0098, 245, 0447, 0464, 0500, and 3) were positive for FMDV RNA, showing cycle threshold (Ct) values ranging from 18.4 to 28.9, indicating active FMDV infection with moderate to high viral RNA loads in the tested oryx, while samples from three other Arabian oryx (female 413 and males 83 and 140) resulted negative. None of the tested Arabian oryx (females 0098, 245, 413, 0447, 0464, 0500, and 3, and males 83 and 140) showed positiveness for PPRV RNA. The sample collected from a sand gazelle male was negative for both FMDV and PPRV antigens.

## 4. Discussion

Foot-and-mouth disease (FMD) is a severe, highly contagious viral disease of livestock that has a significant economic impact. FMD is an OIE-listed disease, affecting cattle, swine, sheep, goats, and other cloven-hoofed ruminants, members of the order *Artiodactyla*. It is a transboundary animal disease (TAD) [8]. There are seven viral serotypes (A, O, C, SAT1, SAT2, SAT3, and Asia1). Recent FMD outbreaks occurred in 2025 in Germany, Hungary, and Slovakia, countries officially free for 50 years, with subsequent application of strict trade restrictions on animals and animal products (including meat and dairy), as per WOAH rules [8], showing the constant potential of the disease for international spread, despite application of biosecurity measures. Oman is not officially FMD-free, as per WOAH, and the disease is present also in the neighboring countries of Yemen and Saudi Arabia, with animal health and international trade implications. In 2023, the risk of FMD introduction and spread in countries in the Near East and West Eurasia was evocated for serotype SAT2 (the same responsible of the outbreak in cattle in Dhofar), reminding us of the susceptibility of wild ruminants [13].

The FMDV serotype O, the most frequent in the region [14], has been recently introduced into the Arabian Peninsula on multiple occasions [15] and it was involved in one outbreak affecting the Arabian oryx in the United Arab Emirates (UAE) in 2015 [14] and in two other outbreaks affecting captive scimitar-horned oryx (*Oryx dammah*), a large Sahelo-Saharan antelope extinct in the wild, in UAE in 2013 and 2015 [16]. In another outbreak affecting scimitar-horned oryx, in UAE in 2013, the FMDV was a serotype A [16]. The Arabian oryx was previously documented as being a spill-over following outbreaks caused by serotype O [17,18]. In the case of the FMD outbreak in Al Wusta, even if the involved serotype was not determined, it is likely that the virus circulating in domestic animals was introduced into the captive oryx population. However, the epidemiological role of the Arabian oryx is still unknown, which calls for further studies [14].

Regarding the confirmatory diagnostics for FMD, virus isolation and histopathological examination represent the gold standard for definitive FMDV diagnosis. In addition, genetic characterization is crucial for understanding the molecular epidemiology and evolutionary dynamics of FMDV, especially in the context of a rare outbreak in a wildlife species such as the Arabian oryx. However, as this outbreak occurred under field conditions in a wildlife sanctuary located in a remote protected area with restricted handling protocols for endangered species, there were significant logistical, biosecurity, and practical limitations. Necessary facilities and cold chain, against high temperatures and rapid decomposition, were unavailable at the outbreak site. This prevented the collection of full necropsy samples and implementation of virus isolation, allowing only the collection of a limited number of samples. Ultimately, due to the acute nature of the outbreak, the focus was on rapid diagnosis and outbreak containment to minimize mortality and further spread.

In addition, sampling was constrained by the need to safely handle wild oryx and minimize stress. Standard sample size calculations (e.g., detecting a 1–5% prevalence at 95% confidence level in large herds) are theoretical and rarely met in such field settings. Samples were deliberately chosen to maximize the chance of detection (e.g., symptomatic or high-risk animals) from animals showing signs and being readily accessible and available at capture. This approach reflects accepted outbreak investigation practice in wildlife under field conditions [19,20,21]. Even with limited sampling, a positive result is highly indicative of an outbreak, and is possibly sufficient in the case of highly diffusive diseases such as FMD.

Nonetheless, despite the absence of virus isolation, several converging lines of evidence collectively support the conclusion of an FMD outbreak in Arabian oryx. Clinical signs in affected animals were highly characteristic of FMDV infection, including salivation and oral erosions, typical of the disease in cloven-hoofed species. The haematological profile was consistent with an acute severe infection of probable viral aetiology, also considering the low values of procalcitonin, which are elevated in case of bacterial or fungal infections.

All samples were processed in accordance with WOAH-recommended protocols and the diagnostic approach was aligned with WOAH recommendations for FMDV detection in wildlife settings, where PCR and NSP serology are acceptable when isolation is impractical [21].

The use of an FMD DIVA ELISA for the detection of NSP antibodies confirmed the exposure to active virus, ruling out vaccination as a cause. FMDV-specific RNA was detected using a RT-qPCR assay targeting the conserved 3D RNA-dependent RNA polymerase gene of the virus. This gene is commonly targeted in diagnostic protocols due to its high sequence conservation across all seven serotypes of FMDV and its suitability for pan-serotype detection. The assay employed follows the WOAH’s recommended protocol, which has been widely validated for sensitivity and specificity in both domestic and wild cloven-hoofed species. Real-time RT-qPCR targeting the conserved 3D gene of FMDV yielded positive results in multiple samples, with Ct values between 18.4 and 28.9, indicating moderate to high viral RNA load. These Ct values are consistent with active FMDV infection. According to the standard interpretation for FMDV RT-qPCR targeting the 3D gene, Ct values ≤ 30 are typically considered strongly positive, as values are inversely proportional to the viral load, particularly when supported by clinical signs and other diagnostic evidence. In addition, the outbreak occurred concurrently with confirmed FMDV circulation in domestic livestock in the surrounding areas of Oman, supporting epidemiological plausibility.

### 4.1. Impact of FMD in Wild Fauna

FMD-susceptible wildlife species in the Near East and west Eurasia include wild sheep and goats, and several species of deer and antelope, including the mountain gazelles and the Arabian oryx [22]. While FMD has been reported in captive Arabian oryx, FMD has not been detected in wild populations [18,22].

FMD occurred in captive populations of Arabian oryx in Bahrain, UAE, and Saudi Arabia with high morbidity and mortality [14,22]. Three FMD outbreaks affecting Arabian Oryx were reported in 2015: one in Saudi Arabia, killing 21 animals out of 32 (65%), and two in UAE, where 12 died out of 44 (27%), and in the other, 30 animals died out of 210 (12.5%). From those reports, it appears that FMD is a deadly disease for the Arabian oryx, with mortality rates ranging between 12.5 and 65% of the affected population [14]. In the FMD outbreaks reported in captive scimitar-horned oryx (*Oryx dammah*) [16], despite high seroconversion rates (up to 78%) and presence of lesions as ulcers on the gum of the upper lip and coronary band characteristic of FMD infection, all animals were in good body condition, their feed intake was normal and they had very low mortality, which is attributable to FMD was noted in the population of nearly 4000 heads.

During the outbreak occurred in Al Wusta Sanctuary, in the daily examination that was ongoing in January 2025, Arabian oryx were identified exhibiting lesions partially compatible with FMD as aphthae and erosions on the oral mucosa, but no animals showed lesions at the level of feet. Sera were analyzed using an ELISA, which specifically measures antibodies directed at FMDV NSP. The results demonstrate that one animal tested was FMDV NSP-seropositive. All other animals were seronegative, probably due to the testing performed before seroconversion in recently infected animals. However, since the test has not been validated for the Arabian oryx species, results should be interpreted with caution. FMDV was also confirmed with real-time RT-PCR for antigen-detection in oral swabs. The results are compatible with an introduction of FMDV and rapid transmission of the virus due to its high contagiousness, which matched the observed clinical cases. Even if it was not possible to determine if FMD accounted for all the recorded mortality, this was considered highly probable also taking into account that no other pathogens were identified. Furthermore, FMD might have played an important role affecting fragile individuals, such as females at the late stage of pregnancy, newborns, or animals with general poor body conditions, eventually being linked to heavy infestations with gastrointestinal parasites such as *Nematodirus* spp. The parasite exams performed in the present study were intended as ancillary data and reported for completeness; their identification was generally in line with expected parasite fauna [23,24]. However, parasites were unlikely to be the main cause of the outbreak, aside from possibly weakening overall body condition, and positive viral test results support our conclusion that the lesions were caused by FMDV.

It has been also noted that the overall health status of the animals decreased before the outbreak in concomitance with changes of feed supply, which was estimated to be of poorer quality in confronting the what was previously available by other feed business operators. Compared to the previous documented mortality rates, related to outbreaks involving limited numbers of animals, the mortality observed in the outbreak occurred in Al Wusta can be considered relatively high and suggests the circulation of a highly virulent FMD strain.

With concern to reem gazelle, native to the Middle East and originally inhabiting the Arabian and Syrian Deserts, it is now primarily found in the Arabian Peninsula, where it persists in the wild as small and isolated herds in Saudi Arabia, UAE, Oman, Yemen, Turkey, Kuwait, Iraq, Jordan, and Syria [25]. The population of mature wild sand gazelles is estimated at around 2150 individuals, classifying the species as vulnerable [26]. A substantially larger number is kept in captivity within reserves and breeding programs, such as Oman’s Yalooni Breeding Center and Al Wusta Sanctuary, as well as notably in Abu Dhabi [26]. In Oman, the Arabian sand gazelle is among the predominant wild ruminant ungulates, and as with other wild ruminants, it is known to be susceptible to FMD. However, contrary to the observations in Arabian oryx, in the present study, only four cases of mortality were reported among the large herd of above 1,000 individuals housed in Al Wusta. In another FMD outbreak which occurred in UAE in 2013, the mortality in this species was very high, with deaths accounting for 663 animals out of a population of 1,095 gazelles (60.7%) [16].

### 4.2. Origine of the Infection in Oryx (Source of the Virus?)

Most of the domestic Omani animals, about the 70%, are raised in Dhofar [27,28], due to the favorable climate and availability of large pastures. The 225,000 bovines and the 187,000 dromedaries constitute the majority of the zootechnic resources of the region, along with less than 100,000 goats and donkeys. Apart from the national immunization plan against FMD, which covers 60% of the domestic large and small ruminant population (dromedaries are excluded), no other prophylactic programs are in place, and farmers are culturally averse. Pastures are freely shared by the different domestic species. Because of close intermingling, there are no barriers preventing disease transmission among animals. Furthermore, farmers often use natural reserves as grazing areas, allowing diseases to spread indirectly from domestic livestock to wildlife. Additionally, the high density of domestic animals increases the epidemiological risk, promoting the circulation of infectious diseases. Movements of live animals from Dhofar towards northern governorates for trade purposes are scarce, but the epidemiological risk exists and only a few animals are sufficient to spread pathogens, especially those with as highly diffusive potential as FMD.

According to the most recent update adopted in 2024 of the WOAH Terrestrial Animal Health Code (Article 8.8.1. General provisions) [8], many different species belonging to diverse taxonomic orders are known to be susceptible to infection with FMD virus. Their epidemiological significance depends upon the degree of susceptibility, the husbandry system, the density and extent of populations, and the contacts between them. For the purposes of the Terrestrial Code, FMD is defined as an infection of the following animals with FMDV: animals of the family *Suidae*; animals of the subfamilies *bovinae*, *caprinae*, and *antilopinae* of the family *Bovidae* and family *Cervidae*; and *Camelus bactrianus*. Amongst the *Camelidae*, Bactrian camels and new world camelids have been shown to be susceptible [9]. According to WOAH, only Bactrian camels (*Camelus bactrianus*) are sufficiently susceptible to have potential for epidemiological significance. As per WOAH rules, dromedaries (*Camelus dromedarius*) are not formally susceptible species to infection with FMDV, while South American camelids are not considered to be of epidemiological significance [8]. Dromedaries are believed to be mostly resistant to clinical FMD, and virus isolation is difficult. Natural and experimental infections occurred in dromedaries; however, they are not believed to play a significant role in transmission to livestock [29]. Experimentally, dromedaries resulted from low susceptibility to inoculation with FMD virus serotype O [30]. Similar results were obtained by investigating the potential role of dromedary camels in the epidemiology of FMD also in Oman. Sera from local dromedaries that grazed with FMD-positive cattle and small ruminants were tested negative for antibodies against serotype O, suggesting that FMDV was not transmitted to dromedary camels [31]. However, in West Africa, serum samples from dromedaries were positive for FMD serotype A, suggesting that dromedaries may be susceptible to FMDV infection, even if likely not playing a significant role in the epidemiology of the disease [32]. In addition, no studies indicated camels to be resistant to the serotype SAT 1, reported to be circulating in Oman in 2023, and antibodies to all seven serotypes of FMDV have been found in domestic dromedary camels [33].

Nevertheless, small ruminants (goats) and especially dromedaries were observed frequently freely roaming very near, and even within, the perimeter of the sanctuary also during the FMD outbreak in oryx, suggesting high risk for naïve susceptible species. Indeed, the occurrence of the FMD outbreak in cattle in Dhofar in 2023, which was reported about 500 km from the Al Wusta Sanctuary, was seriously taken into account by the managers of the center. This motivated a subsequent vaccinal program to prevent the risk of diffusion to oryx. However, while mortality was expected among unvaccinated animals (46.46%), despite the vaccinal status, the 38.49% of dead animals were found to be vaccinated against FMD serotypes A, O, and SAT 1. It should be emphasized that the immunity provided by commercially available FMD vaccines is short and in order to obtain an effective protection of the animals, protocols require a second inoculation after 3 to 4 weeks, followed by a booster every six months. In addition, the specific circulating strains should be considered [14]. Another issue that appeared during the vaccination plan undertaken in the oryx population in Al Wusta was the handling of animals. In particular, the vaccination was given essentially to males, and females were only partially immunized since most of the adult females were pregnant and the risk of abortion due to stress caused by handling was considered high. This was reflected in the mortality rates among unvaccinated animals: the majority (96% of the animals for which the gender was reported) were females. In deaths among vaccinated animals, 75% were males and only 25% were females, simply because only a few females could be vaccinated. The various requirements and practical problems related to the handling of the animals constitute a challenge to effectively protect wildlife species.

### 4.3. Perspectives

A key recent milestone in advancing conservation efforts is Oman’s future-oriented strategy, “Oman 2040” (Vision 2040). Prepared in 2019 under the Royal Directives of His Majesty Sultan Qaboos bin Said, this comprehensive 20-year, multisector plan outlines the nation’s strategic priorities and directions for sustainable development. The defined priorities include environment and natural resources, targeting the inclusion of the Sultanate within the 20 best virtuous countries, in terms of environmental performance indicators, by 2040. This calls the full engagement of the Environment Authority of the Sultanate, established in 2020, which is responsible for the protection and management of the wild fauna patrimony, particularly vulnerable and endangered species, also by research, awareness, and repopulation projects.

Nowadays, Oman remains a land of untamed, largely undiscovered natural beauty. Only a few data are available on wild animals, provided by some field reports and scientific studies. Only a few studies were focused to the Arabian oryx [1,2,7], with very limited information on diseases affecting the species. The present findings highlight not only the risk of FMD or other dangerous pathogens incurred in captive facilities in Oman and other neighboring countries in the Arabian Peninsula, but also underline the necessity to improve biosecurity and prevention by strengthening management standards and knowledge. Improved management requires the availability of adequate facilities. It is of the utmost importance for clinical therapies, surgical interventions, and rapid diagnostic testing (facilities for clinical examinations, treatments, surgery, necropsies, laboratory, and adequate equipment) for effective assistance to captive animals. This should be flanked by training the staff in the best practices. Improved knowledge requires design and implementation of research projects. The complexity of planned activities and expected results dictate the provision of dedicated human resources, equipment, and consumables. The objective of such activities should focus on the better understanding of animal health in wildlife. Such knowledge will allow for the improvement in the management of captive wild fauna, with beneficial effects on health and welfare, reducing diseases and mortality and sustaining repopulation strategies, taking into account the interconnection with domestic animals and related health risks.

## 5. Conclusions

Deserts, majestic mountains, and green landscapes are all part of the Sultanate of Oman, a small country with an abundance of biodiversity, a remarkable natural patrimony that should be preserved. In Oman, historically, the Asiatic lion (*Panthera leo persica*, Meyer, 1826) was also present [34]. The region experienced serious changes that affected the fauna, particularly in the context of the last century. The disappearance of selected species, such as the Arabian oryx (*Oryx leucoryx*) in 1972 [2], and the Asiatic cheetah (*Acinonyx jubatus venaticus*, Griffith, 1821) in 1977 [35], were indicative of these marked changes and serious threats affecting the natural environment of the country. Poaching was certainly the most important threat for wild fauna. In Oman, poaching not only led to the extinction of the oryx, but even hampered repopulation programs, especially during a period of heavy oryx hunting in 1996–1997. Despite that as per Ministerial Decision, the hunting of all species of the wild has been banned since 1993 [36], reports of law infringements [37] call attention to the threat to wildlife. However, even if the problem of uncontrolled hunting, as in the past, is still a reality and deserves efficient preventive measures, such as active monitoring of the territory by rangers and other public forces, risks related to animal health, as demonstrated by the present study, should be carefully taken into account and prevented with adequate resources and trained staff. The survival of endangered wildlife and the achievement of Oman’s Vision 2040 program target can only be ensured by utilizing rational planning, research implementation, and natural resource management.

## Figures and Tables

**Table 1:** Arabian oryx population distributed in four different herds at the Al Wusta Sanctuary, Sultanate of Oman. A total of 669 oryx were reported from the four breeding areas on the 1 January 2025.

Herd/Enclosure	Number of Animals	Adults		Calves		Gender		
		Males	Females	Males	Females	Males	Females	%
Maha 1	163	163	0	0	0	163	0	24.36
Maha 2	232	53	171	3	5	56	176	34.68
Maha 3	194	46	144	0	4	46	148	29.00
HP	80	50	30	0	0	50	30	11.96
Total	669	312	345	3	9	315	354	100

**Table 2:** Vaccinal campaign against FMD applied on the Arabian oryx population at the Al Wusta Sanctuary, Sultanate of Oman, from the 26 December 2023 to the 22 January 2024, following the reported outbreak in cattle that occurred in Dhofar in January 2023. Each animal was immunized with 2 mL subcutaneously (SC) with a polyvalent inactivated vaccine against FMD serotypes O, A, and SAT 2, and commercialized for cattle, sheep, and goats (Vet. Ser. and Vacc. Res. Inst., Cairo, Egypt). * Animals not protected for FMD.

Vaccination	Number of Animals	Gender		
		Males	Females	%
Vaccine for FMD combined with enterotoxaemia	336	274	62	65
Vaccine for FMD combined with enterotoxaemia and hemorrhagic septicemia	129	89	40	25
Vaccine for other diseases only *	52	52	0	10
Total vaccinated	517	415	102	100

**Table 3:** Vaccinal status of dead Arabian oryx (n 226) reported from 8 January to 4 February 2025 at the Al Wusta Sanctuary, Sultanate of Oman. * Animals not protected for FMD.

Vaccination	Number of Animals	Gender			
		Males	Females	Not Reported	%
Vaccine for FMD combined with enterotoxaemia	60	48	12	0	26.55
Vaccine for FMD combined with enterotoxaemia and hemorrhagic septicemia	27	17	10	0	11.94
Total vaccinated for FMD	87	65	22	0	38.49
Vaccine for other diseases only (four enterotoxaemia, five not specified) *	9	8	1	0	3.98
Total vaccinated	96	73	23	0	42.48
Unvaccinated *	105	3	77	25	46.46
Not reported	25	6	18	1	11.06
Total dead animals February 2025	226	82	118	26	100

**Table 4:** Values and means for various hematological and serum biochemical parameters in adults Arabian oryx (*Oryx leukoryx*) compared to values reported from apparently healthy animals referred to as normal reference limits [10,11,12]. Numbers indicated in bold: abnormal values. ND: not determined.

**Hematological Parameters**								
**Parameter (Unit)**	**F 405**	**F 400**	**F 0994**	**F 472**	**M Unt.**	**Mean**	**Range**	**Normal**
WBC (10^3^/µL)	**7.56** (−)	**3.73** (−)	**4.48** (−)	**6.65** (−)	**8.24** (−)	6.13	3.73–8.24	11–14
RBC (10^6^/µL)	11.03	**8.79** (−)	**8.60** (−)	10.48	**8.01** (−)	9.38	8.01–11.03	9–17
Hb (g/dL)	15.8	**11.9** (−)	**12.8** (−)	**14.5** (−)	**11.7** (−)	13.34	11.7–15.8	15–17
HCT (%)	**54.5**	**41.2**	**44.0** (−)	50.3	**40.6** (−)	46.12	40.6–54.5	45–52
MCV (fL)	49.4	46.9	**51.2** (+)	48.0	**50.7** (+)	49.24	46.9–51.2	44–50
MCH (pg)	14.3	13.5	14.9	13.8	14.6	14.22	13.5–14.9	10–27
MCHC (g/dL)	**29.0** (−)	**28.9** (−)	**29.1** (−)	**28.8** (−)	**28.8** (−)	28.92	28.8–29.1	31–34
PLT (10^3^/µL)	**169** (−)	**59** (−)	**33** (−)	**53** (−)	**360** (+)	134.8	33–360	183–327
RDW-SD (fL)	28.1	ND	29.9	ND	26.8	28.26	26.8–29.9	
RDW-CV (%)	23.3	22.6	21.4	23.9	19.4	22.12	19.4–23.9	
MPV (fL)	8.0	8.4	7.8	7.5	9.1	8.16	7.5–9.1	
P-LCR (%)	10.9	9.5	5.0	6.9	21.7	10.08	5.0–21.7	
PCT (%)	0.14	0.05	0.03	0.04	0.33	0.11	0.03–0.33	
NRBC (10^3^/µL)	0.01 (0.1%)	0.01 (0.3%)	0.01 (0.2%)	0.01 (0.2%)	0.01 (0.1%)	0.01 (0.18%)	0.01 (0.1–0.3%)	
Neutrophyls (10^3^/µL)	0.27 (3.5%)	0.68 (18.3%)	0.20 (4.5%)	0 (0%)	0.04 (0.5%)	0.23 (5.36%)	0–0.68 (0–18.3%)	0.55–2.68 (56.5–75.2%)
Lymphocytes (10^3^/µL)	6.79 (89.8%)	2.82 (75.6%)	3.88 (86.6%)	6.28 (94.4%)	7.74 (93.9%)	5.50 (88.06%)	2.82–7.74 (75.6–94.4%)	0.70–0.79 (20.5–28.4%)
Monocytes (10^3^/µL)	0.36 (4.8%)	0.21 (5.6%)	0.35 (7.8%)	0.31 (4.7%)	0.42 (5.1%)	0.33 (5.6%)	0.21–0.42 (4.7–7.8%)	0.03–0.04 (0.78–1.0%)
Eosinophyls (10^3^/µL)	0.08 (1.1%)	0 (0%)	0 (0%)	0 (0%)	0 (0%)	0.01 (0.22%)	0–0.08 (0–1.1%)	0–0.10 (0–2.86%)
Basophyls (10^3^/µL)	0.06 (0.8%)	0.02 (0.5%)	0.05 (1.1%)	0.06 (0.9%)	0.04 (0.5%)	0.04 (0.76%)	0.02–0.06 (0.5–1.1%)	0–0.06 (0–2.42%)
**Serum Biochemical Parameters**								
**Parameter (Unit)**		**F 405**	**F 400**	**F 0994**	**F 472**	**Mean**	**Range**	**Normal**
Albumin (g/dL)		**3.84**	**2.68**	**2.56**	**3.90**	3.24	2.56–3.90	4.5–5.2
Alkaline phosh (U/L)		**135.60**	**79.36**	**109.34**	**142.17**	116.61	79.36–142.17	111–115
Bilirubine direct (mg/dL)		**0.35**	**0.90**	**1.85**	**0.67**	0.94	0.35–1.85	0.008–0.01
Bilirubine total (mg/dL)		0.37	**1.14**	**2.89**	**0.80**	1.3	0.80–2.89	0.03–0.36
CK NAK (U/L)		**691.72**	**783.10**	**902.24**	**788.88**	791.48	691.72–902.24	303–367
Creatinine (mg/dL)		**1.86**	1.37	1.25	**1.83**	1.57	1.25–1.86	0.92–1.38
Gamma GT (U/L)		8.71	11.61	14.30	9.33	10.98	8.71–14.30	
Glucose (mg/dL)		**80.24**	**77.81**	**78.51**	**96.39**	83.23	77.81–96.39	119–150
GOT AST (U/L)		60.86	**211.87**	ND	**83.02**	118.58	60.86–211.87	47–63
GPT ALT (U/L)		36.50	**49.34**	**109.03**	43.86	59.68	36.50–109.03	35–45
Magnesium (mg/dL)		1.7	1.6	1.6	**1.9**	1.7	1.6–1.9	1.69–3
Urea (mg/dL)		**36.3**	**147.4**	**42.3**	**58.9**	71.22	36.3–147.4	20–25
Calcium (mg/dL)		**9.46**	8.34	**7.47**	8.73	8.5	7.47–9.46	8–9
Copper (µg/dL)		88	77	77	95	84.25	77–95	
Iron (µg/dL)		78.18	36.65	49.14	71.48	58.86	36.65–78.18	
Phosphorus (mg/dL)		**7.67**	9.96	9.04	**3.91**	7.64	3.91–9.96	8–10

**Table 5:** Results of serological screening for antibodies to contagious bovine pleuro polmonitis (CBPP), Johne’s disease (JD), Peste des petits ruminants virus (PPRV), and foot-and-mouth disease virus (FMDV), and antigenic detection for PPRV and FMDV in animals from Al Wusta Sanctuary of the Sultanate of Oman. Ab: antibodies; and NE: not executed, in certain cases due to hemolysis.

Animal Species	Gender	Ear Tag Number	Tests					
			CBPP Ab	JD Ab	PPRV Ab	PPRV RNA	FMDV Ab	FMDV RNA
Arabian oryx	Female	0094	NE	NE	NE	NE	NE	NE
Arabian oryx	Female	0098	NE	Negative	Negative	Negative	Negative	**Positive**
Arabian oryx	Female	0447	NE	Hemolysis	Hemolysis	Negative	Hemolysis	**Positive**
Arabian oryx	Female	0464	NE	NE	NE	Negative	NE	**Positive**
Arabian oryx	Female	0500	NE	Hemolysis	Hemolysis	Negative	Hemolysis	**Positive**
Arabian oryx	Female	0994	Negative	NE	NE	NE	NE	NE
Arabian oryx	Female	3	NE	NE	Doubtful	Negative	**Positive**	**Positive**
Arabian oryx	Female	245	NE	NE	NE	Negative	NE	**Positive**
Arabian oryx	Female	400	Negative	NE	NE	NE	NE	NE
Arabian oryx	Female	405	Negative	NE	NE	NE	NE	NE
Arabian oryx	Female	413	NE	Negative	Negative	Negative	Negative	Negative
Arabian oryx	Female	472	Negative	NE	NE	NE	NE	NE
Arabian oryx	Male	83	NE	Negative	Negative	Negative	Negative	Negative
Arabian oryx	Male	140	NE	Negative	Negative	Negative	Negative	Negative
Arabian oryx	Male	Untagged	Negative	NE	NE	NE	NE	NE
Sand gazelle	Male	Untagged	NE	NE	NE	Negative	NE	Negative

## Data Availability

The article includes original contributions of the study and additional queries can be addressed to the corresponding author.

## Supplementary Materials

The following supporting information can be downloaded at: https://www.mdpi.com/article/10.3390/ani15162389/s1, Table S1: Mortality in animals at Al Wusta Sanctuary of the Sultanate of Oman, from 8 January to 4 February 2025. Out of 669 presents in the sanctuary at the beginning of the outbreak (1 January 2025) (M 312; F 345; Calves 12) subdivided in 4 herds (in 1 only males 163; in 2 M 53 F 171 C 8; in 3 M 46 F 144 C 4 in 4 M 50 F 30), a total of 226 (33.78%) dead oryx were reported (images of ear tag numbers available for most of animals). In the same period were reported also deaths in 4 reems and 1 blackbuck.

## References

[1] Hetem R.S., Strauss W.M., Ick L.G., Maloney S.K., Meyer L.C.R., Fuller A., Shobrak M., Mitchell D. (2012). Selective brain cooling in Arabian oryx (*Oryx leucoryx*): A physiological mechanism for coping with aridity?. J. Exp. Biol..

[2] Spalton J.A. (1993). A brief history of the reintroduction of the Arabian oryx *Oryx leucoryx* into Oman 1980–1992. Int. Zoo Yearb..

[3] IUCN *Oryx leucoryx*. SSC Antelope Specialist Group (2017). IUCN Red List of Threatened Species.

[4] Patzelt A. (2015). Synopsis of the Flora and Vegetation of Oman, with Special Emphasis on Patterns of Plant Endemism. Abh. Braunschw. Wiss. Ges..

[5] UNESCO (2022). Arabian Oryx Sanctuary.

[6] UNESCO (2007). Oman’s Arabian Oryx Sanctuary: First Site Ever to Be Deleted from UNESCO’s World Heritage List.

[7] Goraya K., Al Rawahi Q., Al Balushi S., Al Saadi H., Al Rahbi S., Al Alawi Z., Hussain M.H., Hussain M. (2021). Postmortem findings in captive sand Gazelle and Arabian Oryx at Al-Wusta Wildlife Reserve, Oman. Pak. J. Zool..

[8] WOAH (2025). Foot and Mouth Disease. Listed Diseases, with Official Disease Status. https://www.woah.org/en/disease/foot-and-mouth-disease/.

[9] Larska M., Wernery U., Kinne J., Schuster R., Alexandersen G., Alexandersen S. (2009). Differences in the susceptibility of dromedary and Bactrian camels to foot-and-mouth disease virus. Epidemiol. Infect..

[10] Vassart M., Greth A. (1991). Hematological and Serum Chemistry Values for Arabian Oryx (*Oryx leucoryx*). J. Wildl. Dis..

[11] Hussein M.F., Al-Jumaah R.S., Homeida A., Alhaidary A.A., Alshaikh M.A., Garelnabi A., Mohamed O., Omer S. (2010). Hemostatic profile, platelets, and blood constituents of the Arabian oryx (*Oryx leucoryx*). Comp. Clin. Pathol..

[12] Eljarah A., Ismail Z.B. (2023). Hematology and serum biochemistry variables in apparently normal Arabian Oryx (*Oryx leucoryx*). Vet. World.

[13] McLaws M., Ahmadi B.V., Condoleo R., Limon G., Kamata A., Arshed M., Rozstalnyy A., Rosso F., Dhingra M. Risk of Foot-and-Mouth Disease SAT2 Introduction and Spread in Countries in the Near East and West Eurasia—FAO Qualitative Risk Assessment, FAO, Rome, Italy 2023. https://openknowledge.fao.org/server/api/core/bitstreams/667b4c4c-b80d-4206-bd5f-d432a02d7387/content.

[14] Lignereux L., Alzahlawi N., Al Kharusi Y., Pesci M.E. (2018). Middle East Arabian Oryx Disease Survey.

[15] Bachanek-Bankowska K., Di Nardo A., Wadsworth J., Mioulet V., Pezzoni G., Grazioli S., Knowles N.J. (2018). Reconstructing the evolutionary history of pandemic foot-and-mouth disease viruses: The impact of recombination within the emerging O/ME-SA/Ind-2001 lineage. Sci. Rep..

[16] Lignereux L., Chaber A.L., Saegerman C., Heath L., Knowles N.J., Wadsworth J., Mioulet V., King D.P. (2020). Foot-and-mouth disease outbreaks in captive scimitar-horned oryx (*Oryx dammah*). Transbound. Emerg. Dis..

[17] Ostrowski S., Anajariyah S. (2003). 2002 Middle East Arabian Oryx Disease Survey.

[18] Frölich K., Hamblin C., Jung S., Ostrowski S., Mwanzia J., Streich W.J., Anajariyah S. (2005). Serologic surveillance for selected viral agents in captive and free-ranging populations of Arabian oryx (*Oryx leucoryx*) from Saudi Arabia and the United Arab Emirates. J. Wildl. Dis..

[19] Eltahir Y.M., Ishag H.Z.A., Wadsworth J., Hicks H.M., Knowles N.J., Mioulet V., King D.P., Mohamed M.S., Bensalah O.K., Yusof M.F. (2024). Molecular Epidemiology of Foot-and-Mouth Disease Viruses in the Emirate of Abu Dhabi, United Arab Emirates. Vet. Sci..

[20] Ryser-Degiorgis M.-P. (2013). Wildlife health investigations: Needs, challenges and recommendations. BMC Vet. Res..

[21] World Organisation for Animal Health (2016). Training Manual on Wildlife Diseases Outbreak Investigations. Fourth Cycle Workshop for OIE National Focal Points for Wildlife. https://www.oie.int.

[22] Weaver G.V., Domenech J., Thiermann A.R., Karesh W.B. (2013). Foot and mouth disease: A look from the wild side. J. Wildl. Dis..

[23] Mohammed O.B., Alagaili A.N., Omer S.A., Hussein M.F. (2012). Parasites of the Arabian Oryx (*Oryx leucoryx*, Pallas, 1777) and Their Prevalence in the Kingdom of Saudi Arabia. Comp. Parasitol..

[24] Pauling C.D., Oller A.R., Jackson V. (2016). Fecal parasite identification by microscopy and PCR in scimitar-horned oryx, *Oryx dammah*, managed at two sites. Int. J. Parasitol. Parasites Wildl..

[25] Mallon D.P., Kingswood S.C. (2001). Antilopes—Enquête Mondiale et Plans D’action Régionaux, Partie 4: Afrique du Nord, Moyen-Orient et Asie. Cartes de Distribution.

[26] IUCN *Gazella marica*. SSC Antelope Specialist Group (2017). IUCN Red List of Threatened Species.

[27] Ministry of Agriculture (2011). Agriculture & Livestock Research—Five-Year Research Strategy 2011–2015.

[28] Food and Agriculture Organization (FAO) Crops and Livestock Products: Oman. FAO STAT, 2021. https://www.fao.org/faostat/en/#data/QCL.

[29] Wernery U., Kaaden O.R. (2004). Foot-and-mouth disease in camelids: A review. Vet. J..

[30] Alexandersen S., Wernery U., Nagy P., Frederiksen T., Normann P. (2008). Dromedaries (*Camelus dromedarius*) are of Low Susceptibility to Inoculation with Foot-and-Mouth Disease Virus Serotype O. J. Comp. Pathol..

[31] Body M.H.H., Al-Senaidi N.Y.A., Al-Subhi A.H.A., Al-Maawali M.G., Ahmed M.S., Hussain M.H. (2019). Foot and mouth disease virus serological study of dromedary camels in Oman. Sci. Tech. Rev..

[32] Ularamu H.G., Wungak Y.S., Lazarus D.D., Woma T.Y., Ehizibolo D.O., Dogonyaro B.B., Bwala D.G., Bakari A.H., Agom D., Onoja M.A. (2015). Serological evidence of foot-and-mouth disease virus (FMDV) in camels (*Camelus dromedaries*) in Nigeria. J. Vet. Med. Anim. Health.

[33] Yousef M.R., Mazloum K.S., Al-Nakhli H.M. (2012). Serological evidence of natural exposure of camels (*Camelus dromedarius*) to foot and mouth disease virus. Vet. World.

[34] Mallon D., Budd K. (2011). Regional Red List Status of Carnivores in the Arabian Peninsula.

[35] Harrison D.L. (1983). The mammal fauna of Oman with special reference to conservation and the Oman Flora and Fauna Survey. J. Oman Stud..

[36] Insall D.H., Mallon D.P., Kingswood S.C. (2001). Oman. Antelopes: North Africa, the Middle East, and Asia. Global Survey and Regional Action Plans.

[37] Vaidya S.K. 6 Suspect Gazelle Poachers Held in Oman. Gulf News 2013. http://gulfnews.com/news/gulf/oman/6-suspect-gazelle-poachers-held-in-oman-1.1204189.

