# Supplementary material for: Wild Fauna in Oman: Foot-and-Mouth Disease Outbreak in Arabyan Oryx (Oryx leucorix)
Source: Animals (Basel). 2025 Aug 14;15(16):2389. doi: 10.3390/ani15162389 (PMC12382891; doi:10.3390/ani15162389)
Supplement: Supplementary file 1 [file animals-15-02389-s001.zip › animals-3733558-supplementary.pdf]

Supplementary Material Table S1. Mortality in animals at Al Wusta Sanctuary of the Sultanate of Oman, from January 8th to February 4th 2025. Out of 669 presents in the sanctuary at the beginning of the outbreak (January 1st, 2025) (M 312; F 345; Calves 12) subdivided in 4 herds (in 1 only males 163; in 2 M 53 F 171 C 8; in 3 M 46 F 144 C 4 in 4 M 50 F 30), a total of 226 (33.78%) dead oryx were reported (images of ear tag numbers available for most of animals). In the same period were reported also deaths in 4 reems and 1 blackbuck.

Animal (ear tag number)	Gender	Vaccine	Note
0002	F	No	
0005	F	No	
0026		No	
0029	F	No	
0045	F	No	
0087	F	No	
0091	F	No	
0095		No	
0097	F	No	
0099	F	NO	M3
07	F	NO	21/01/25 M2
028	M	0028 Yes Enterotoxaemia, FMD	Adult L- yellow (y) M1
029	F	NO	Adult R-y M2
0100	M	Yes Other	Not protected for FMD
0104		No	
0107	F	No	
0114	F	No	316 L-y old
0119	F	No	
0133	F	No	old
0134	F	No	old
0137	F	No	
0150	F	No	
0153	M	Yes Enterotoxaemia, FMD	11/01/25 Old L-y M3
0162	M	Yes Enterotoxaemia, FMD	
0174	M	Yes Enterotoxaemia, FMD	

0191	F	No	
0193	F	No	
0194	F	No	
0196	F	NO	Adult R-y M2
0231	M	Yes Enterotoxaemia, FMD	
0237	M	No	
0242	M	Yes Enterotoxaemia, Hemorrhagic septicemia, FMD	
0244	F	No	
0249	M	Yes Enterotoxaemia, Hemorrhagic septicemia, FMD	Clinical disease (seak, isolate, loss of activity)
0280	M	Yes Enterotoxaemia, FMD	
0281	F	No	
0283	M	Yes Enterotoxaemia, FMD	
0290		No	
0294		No	
0301	M	Yes Enterotoxaemia, FMD	
0304	M	Yes Enterotoxaemia, Hemorrhagic septicemia, FMD	
0332		No	
0334		No	
0339	M	Yes Enterotoxaemia, FMD	
0342	F	No	
0346	M	Yes Other	Not protected for FMD Therapy
0347	F	No	
0364	F	No	Old
0365	M	Yes Enterotoxaemia, Hemorrhagic septicemia, FMD	
0400	F	No	
0410	F	No	Therapy
0423	M	Yes Enterotoxaemia, FMD	

0424	F	No	
0425		No	
0428	F	No	Clinical disease (very weak, dystocia)
0433	F	No	
0437	M	Yes Enterotoxaemia, FMD	
0442	M	Yes Enterotoxaemia, FMD	
0443	M	Yes Enterotoxaemia, Hemorrhagic septicemia, FMD	
0447	F	Yes Enterotoxaemia, Hemorrhagic septicemia, FMD	SAMPLED (447 F vaccinated) 15/1/25
0453	F	No	
0461	F	No	
0464	F	Yes Enterotoxaemia, FMD	SAMPLED (464 F vaccinated) 15/1/25
0465	M	Yes Enterotoxaemia, FMD	
0468	M	Yes Enterotoxaemia, FMD	
0469		No	
0472	F	No	
0473	F	No	
0476	F	No	
0496	F	Yes Enterotoxaemia, Hemorrhagic septicemia, FMD	M3
0499	M	Yes Enterotoxaemia, FMD	
0500	F	Yes Enterotoxaemia, FMD	SAMPLED (500 F vaccinated)
0520	F	Yes Enterotoxaemia, Hemorrhagic septicemia, FMD	
0530	F	No	
0534	M	No	
0536	M	Yes Enterotoxaemia, FMD	
0590	F	No	
0593	F	No	

0605		No	
0659	F	No	
0660	F	No	
0661	F	No	
0667	F	No	
0668	M	Yes Enterotoxaemia, FMD	Therapy
0674		No	
0686	F	No	
0688		No	
0691	F	No	Therapy
0709	M	Yes Enterotoxaemia, FMD	
0711	M	Yes Enterotoxaemia, Hemorrhagic septicemia, FMD	
0713	M	Yes Other	Not protected for FMD
0714		No	
0715	F	No	Old
0718		No	
0726	M	Enterotoxaemia	Not protected for FMD Adult L -y M1
0730	F	No	
0733	M	Yes Enterotoxaemia, FMD	
0741	F	No	
0744	F	No	
0748	F	No	
0752	F	No	
0758	F	No	Therapy
0759	M	Yes Enterotoxaemia, Hemorrhagic septicemia, FMD	
0762		No	
0767	M	Yes Enterotoxaemia, FMD	
0770	F		
0772	F	No	Therapy

0777	M	Yes Enterotoxaemia, FMD	Therapy
0788	F	No	
0803	F	No	
0804	F	No	
0903	M	Yes Other	Not protected for FMD
0904		No	
0905	M	Yes Enterotoxaemia, FMD	
0907	M	Yes Enterotoxaemia, FMD	
0909	F	No	
0912	F	No	Therapy
0915	M	Yes Enterotoxaemia, Hemorrhagic septicemia, FMD	
0916		No	
0924		No	
0925		No	
0926	F	No	
0929		No	
0932	F	No	Therapy
0934	M	Yes Enterotoxaemia, Hemorrhagic septicemia, FMD	
0936	F	Enterotoxaemia	Not protected for FMD Adult R-y M3
0939	F	No	therapy
0942		No	
0947	F	No	
0949	M	Yes Enterotoxaemia, FMD	
0950	M	Yes Enterotoxaemia, FMD	
0965	M	Yes Enterotoxaemia	Not protected for FMD
0969	F	No	
0973	F	No	
0984	F	No	
0994	F	No	SAMPLED

0998	F	No	
3	F	No	(mounsa) SAMPLED
23		No	
38		No	
46	F	No	
53	F	Yes Enterotoxaemia, FMD	
68	F	Yes Enterotoxaemia, FMD	
70	M	Yes Enterotoxaemia, FMD	
72	F	Yes Enterotoxaemia, FMD	
76	F	Yes Enterotoxaemia, FMD	
88	M	Yes Enterotoxaemia, FMD	
96	M	Yes Enterotoxaemia, FMD	
100	F	No	
103	M	No	
106	M	Yes Enterotoxaemia, FMD	Sub-adult L106/y M1
115	M	Yes Enterotoxaemia, FMD	
120	F	Yes Enterotoxaemia, Hemorrhagic septicemia, FMD	
127	M	Yes Enterotoxaemia, FMD	33127 M1
128	M	Yes Enterotoxaemia, Hemorrhagic septicemia, FMD	
129	F	Yes Enterotoxaemia, FMD	
139	F	Yes Enterotoxaemia, Hemorrhagic septicemia, FMD	Adult R-y M3
160	F	Yes Enterotoxaemia, Hemorrhagic septicemia, FMD	
161	M	Yes Enterotoxaemia, Hemorrhagic septicemia, FMD	
170	M	Yes Enterotoxaemia, FMD	
172	F	Yes Enterotoxaemia, Hemorrhagic septicemia, FMD	16/1/25
173	M	Yes Enterotoxaemia, Hemorrhagic septicemia, FMD	
175	M	Yes Enterotoxaemia, FMD	L-y

181	M	Yes Enterotoxaemia, Hemorrhagic septicemia, FMD	
184	M	Yes Enterotoxaemia, FMD	
203		No	
237	M	Yes Enterotoxaemia, Hemorrhagic septicemia, FMD	
240	M	Yes Enterotoxaemia, Hemorrhagic septicemia, FMD	Therapy
243	M	Yes Enterotoxaemia, Hemorrhagic septicemia, FMD	
245	F	Yes Enterotoxaemia, FMD	SAMPLED
280	M	Yes Enterotoxaemia, FMD	
302	F	Yes Enterotoxaemia, FMD	M3
305	M	Yes Enterotoxaemia, FMD	
306	M	Yes Enterotoxaemia, FMD	
308	M	Yes Enterotoxaemia, FMD	
311	M	Yes Enterotoxaemia, FMD	
312	M	Yes Enterotoxaemia, FMD	
314	M	Yes Enterotoxaemia, FMD	Old Sp L-y
315	M	Yes Enterotoxaemia, FMD	
316	M	Yes Enterotoxaemia, FMD	
317	M	Yes Enterotoxaemia	Not protected for FMD
322	F	Yes Enterotoxaemia, Hemorrhagic septicemia, FMD	
334	F	Yes Enterotoxaemia, FMD	
335	M	Yes Enterotoxaemia, FMD	
338	M	Yes Enterotoxaemia, FMD	
349	F	No	
352	M	Yes Enterotoxaemia, FMD	
387	M	Yes Enterotoxaemia, FMD	
400	F	Yes Enterotoxaemia, FMD	SAMPLED
410	F	Yes Enterotoxaemia, FMD	L-410/orange

429	F	Yes Enterotoxaemia, Hemorrhagic septicemia, FMD	
450	F	No	Therapy
469	F	No	
473	F	Yes Enterotoxaemia, Hemorrhagic septicemia, FMD	M4
488	M	Yes Enterotoxaemia, Hemorrhagic septicemia, FMD	
760	F	NO	M4
971		No	
1000	M	Yes Enterotoxaemia, FMD	
7660	F	No	
9042	F	No	
33199	F	No	
K2000	F	No	
Untagged	M		Juvenile M3
Untagged	M		
Untagged	M		
Untagged	M		HP1
Untagged	M		Calf
Untagged	M		Calf
Untagged	F		calf
Untagged	F		Calf
Untagged	F		Calf
Untagged	F		New kid
Untagged	F		New kid
Untagged	F		New kid
Untagged	F		New kid
Untagged	F		old
Untagged	F		juvenile
Untagged	F		adult
Untagged	F		adult

Untagged	F		adult
Untagged	F		adult
Untagged	F		adult
Untagged	F		
Untagged	F		
Untagged	F		therapy
Untagged	F		Calf
203 (Reem)	M	No	Reem
33949 (Reem)	F	No	Reem
Untagged (Reem)	F		Reem
Untagged (Reem)			Reem
Untagged (Blackbuck)			Blackbuck (HP)
